# A case of tracheotomy using the Ex Utero Intrapartum Treatment (EXIT) procedure in cooperation with multiple professions

**DOI:** 10.20407/fmj.2021-030

**Published:** 2023-05-09

**Authors:** Yasunori Asai, Hisayuki Kato, Kanetaka Horibe, Yusuke Hiei, Ichiro Tateya

**Affiliations:** Department of Otolaryngology-Head and Neck Surgery, Fujita Health University, School of Medicine, Toyoake, Aichi, Japan

**Keywords:** Ex utero intrapartum treatment (EXIT), Airway management, Tracheotomy, Cystic lymphangioma

## Abstract

In this study, we report a case of tracheotomy using the ex utero intrapartum treatment (EXIT) procedure in a fetus that was pointed out as having bilateral giant cervical cysts at prenatal diagnosis and whose postnatal airway occlusion was predicted. The subject was a female aged 35. She was diagnosed with polyhydramnios at 28 weeks of pregnancy. The fetus was found to have a giant cervical cyst before she was referred to the department of obstetrics and gynecology of our hospital. On the second day of 37 weeks of pregnancy, oral tracheal intubation was attempted on the fetus using the EXIT procedure after the caesarean operation, but intubation was difficult resulting in a tracheotomy. The oxygenation of the fetus during the operation was maintained well without any postoperative complication. Postnatal fetal airway occlusion is a critical incident which may lead to the death of a fetus. It was assumed, however, that the airway management under the same procedure was completed by the preoperative detailed simulation with the staff of the departments of anesthesiology, obstetrics and gynecology and pediatrics as well as the operating room personnel.

## Introduction

Ex utero intrapartum treatment (EXIT) procedure is a technique to treat a fetus while maintaining oxygenation without the separation of the fetal umbilical cord which is delivered through the caesarean operation during birth. This technique is said to be an effective method of airway management for patients with difficulty in airway management after birth.^1–5^ This study will report a case where the subject was a fetus with predicted postnatal airways occlusion due to giant cervical lymphangioma having difficulty in tracheal intubation under the EXIT procedure. Tracheotomy was conducted in cooperation with multiple professions, resulting in the safe airway management.

## Case

*A 35 years-old female*, 153 cm in height and 56 kg in weight. Three pregnancy experiences and one birth experience.

*Past history*: No special findings

*Current history*: After natural pregnancy, the female was indicated to have polyhydramnios by a local doctor at the 28th week of pregnancy. So, she was referred to and visited a general hospital at the 30th week of pregnancy. At the hospital, a transvaginal ultrasonography detected a cystic mass at the fetal cervix. At the 34th week of pregnancy, the patient was referred to our department of obstetrics and gynecology for the purpose of perinatal management.

**Figure 1:**
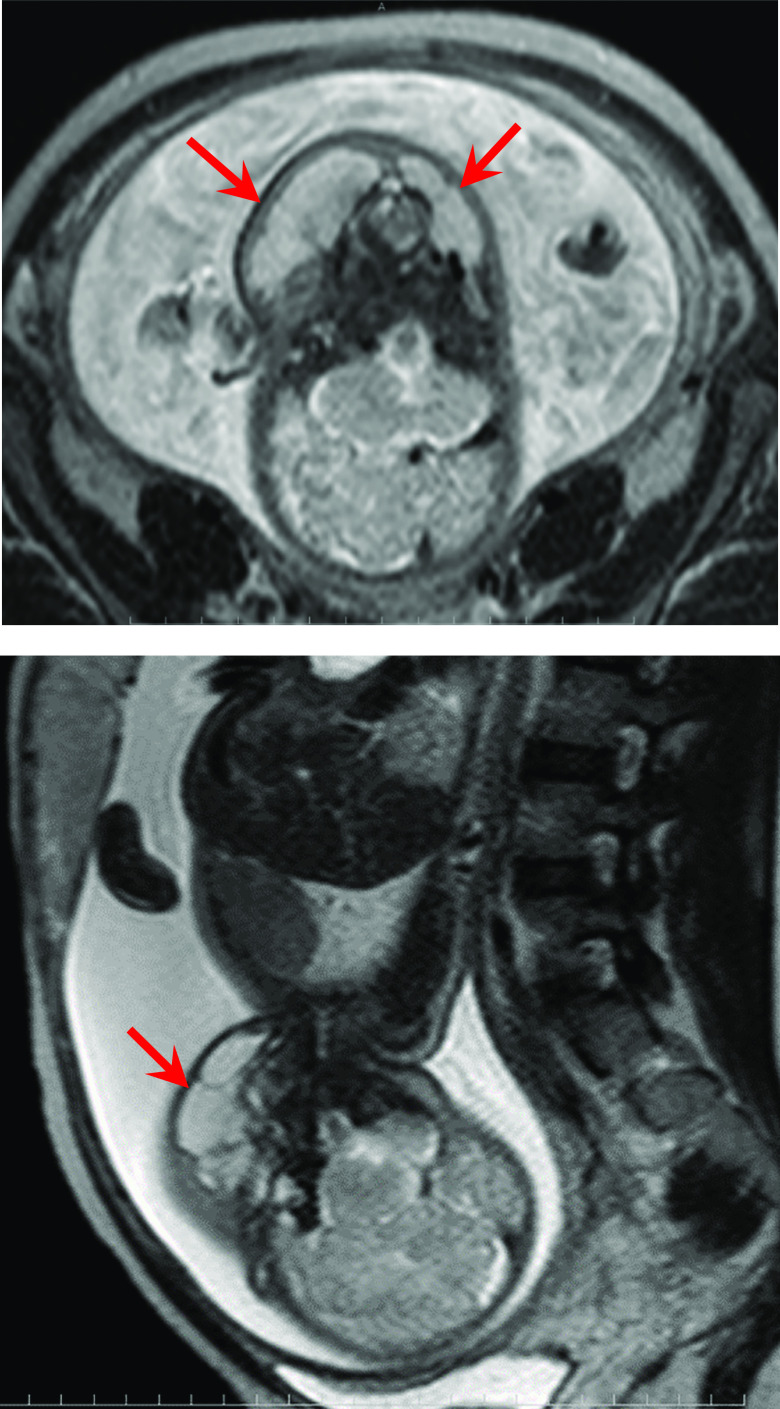
T2-weighted MRI T12-weighted Image with high intensity on the second day of the 35th week of pregnancy (A: T2 Axial, B: T2 Sagital) Multilocular cystic mass lesions having almost internal uniformity were observed in the bilateral cervixes

*Imaging findings*: At the first diagnosis (the first day of the 34 weeks of pregnancy), only a cystic lesion having a major diameter of 10 mm was found during the fetal ultrasonographic examination and diagnosed as being capable of outpatient follow-up. At the second diagnosis (the second day of the 35 weeks of pregnancy), the rapid enlargement of bilateral cervical mass lesions (right: 39×13×28 mm, left 46×14×33 mm) was observed, so the female was immediately admitted to our hospital. The fetal MRI after admission, revealed bilateral-cervical multilocular cystic lesions of which the insides were almost uniform, with low intensity on the T1-weighted image and high intensity on the T2-weighted image (Figure 1). From the image findings, the fetus was diagnosed with cervical cystic lymphangioma. The image findings showed no obvious occlusion of the airway and gastrointestinal tract.

*Treatment strategy*: A joint conference was held on the response during delivery among the personnel of the departments of obstetrics and gynecology, pediatrics, pediatric surgery, anesthesiology, radiology, otolaryngology, as well as nurses and a midwife. As a result, the cause of polyhydramnios was judged to be dysphagia due to cystic compression, and the upper airway stenoses just after birth was predicted, leading to the consideration of an indication of the airway management using the EXIT procedure.

As the method of the airway management of the fetal, it was decided that the following procedures were to be used: first, tracheary intubation would be conducted by the pedologist and the anesthetist, and second, in the case of difficult incubation, tracheotomy was performed by the otolaryngologist. The period for tracheary intubation was set up to 20 minutes, and that of the EXIT procedure was set to 30 minutes, A joint simulation was implemented on the careful confirmation of the equipment to be used, the arrangement of the personnel, the anesthetic induction method, and the airway management, after which delivery using the EXIT procedure was scheduled on the second day of the 37th week of pregnancy.

*Anesthetic management and surgery*: In surgery, the obstetrician performed the caesarean operation under the general anesthesia. The total intravenous anesthesia (TIVA) was used as the general anesthesia, and sevoflurane inhalation and continuous drip infusion of nitroglycerin (Millisro^®^) was conducted for uterine relaxation. The fetal position was confirmed after the rupture of the membrane, the fetal head and right upper arms were delivered. Next, the fetus was equipped with a monitor to check the fetal heartbeat, umbilical blood flow by ultrasonography, and the condition of the placenta, and the delivered fetal head and body trunk were fixed. The tracheary intubation with the airway scope was used by the pedologist, but it was difficult to pass the tracheal tube, and the insertion of it was found to be difficult. Then, the tracheary intubation using the laryngoscope was tried by the pedologist, it was difficult to observe the glottal, and the insertion was also found to be difficult.19 minutes after starting the EXIT procedure, we decided to maintain the airway using the tracheotomy by an otolaryngologist.

**Figure 2:**
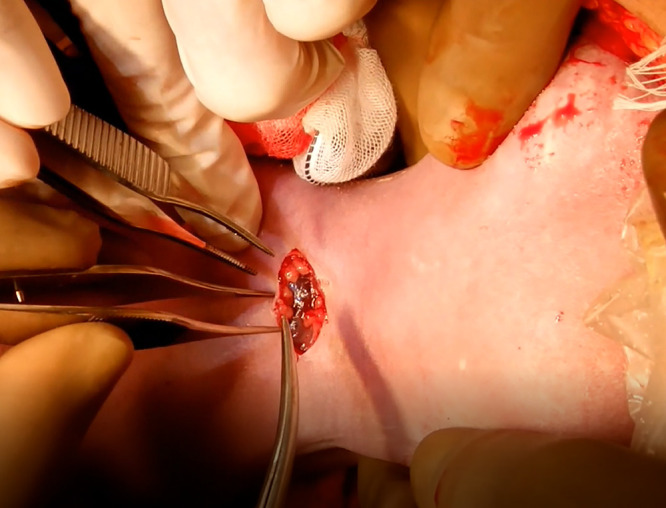
Image of the tracheotomy shown in the photograph The fetal cervical skin was resected transversely, and a cystic mass was exposed.

**Figure 3:**
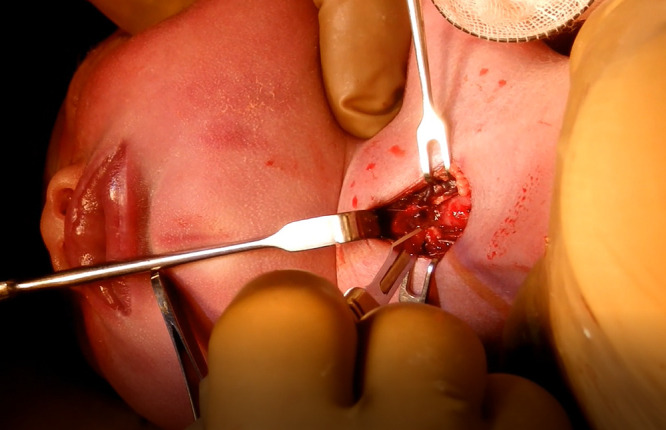
Image of tracheotomy during the operation The cyst was peeled and lifted toward the head, the frontalis muscle group was pulled to the outside to expose the windpipe, and tracheotomy was implemented.

The median skin of the fetal cervix was resected transversely by 20 mm, and a dark-blue cystic mass was exposed (Figure 2). The cysts were peeled and lifted from the surrounding tissue toward the head to check the cricoid cartilage and the tracheary on palpation. Furthermore, while the connective tissue was cauterized appropriately, the anterior cervical muscle was peeled in the median line to expose the airway (Figure 3). The thyroid gland was not observed in the operative field. The second and third tracheal rings were incised in the inverted V-shape to insert the intubation tube of 3.5 mm in length, and the umbilical cord was severed after confirmation of ventilation. The total operation period was 86 minutes, the anesthesia period was 151 minutes, the EXIT procedure period was 31 minutes (from the delivery of the fetus to the separation of the umbilical cord), the tracheal incision period was 11 minutes, and the amount of bleeding including the amnion fluid was 965 g.

*Postoperative course:* The mother had a satisfactory postoperative course not different from the normal postoperative caesarean operation and discharged on the seventh days after the surgery. On the seventh day after the operation, the first cannula replacement was performed in our department, and the suture around the stoma was removed on the 14th day after the operation. In addition, the fetal cystic lymphangioma was treated by conducting the sclerotherapy with the administration of OK-432 (PICIBANIL^®^) three times (1st: 0.8 KE, 2nd: 1.0 KE, 3rd: 1.0 KE) , and the fetus was discharged on the 40th day after the operation. 2.8 years after the surgery, no cannula trouble was found, and the closure of the stoma will be planned after the end of the treatment of lymphangioma in the future.

## Discussion

The EXIT procedure was reported by Kelly et., al. in 1990 as a method for airway management by keeping the fetal placental circulation for a fetus with upper-respiratory-tract occlusion caused by a cervical mass.^6^ The method was improved by Harrison et al. as a method of release just before delivery for a fetus with congenital diaphragmatic hernia after the airway occlusion occurred in the womb.^7^ Thereafter, as a result of indications of the method, it was established as a life-saving measure for a fetus with advanced upper airway stenosis caused by, for example, giant cervical masses and congenital laryngeal occlusion.^1^ Novoa, et al. reported that as of 2020, 235 cases underwent the EXIT procedure, tracheotomy was implemented to 50 cases among them.^8^ In addition, they reported that indications were found in 84 cases of cervical teratoma, 60 cases of lymphangioma, 13 cases of cystic hygroma, 15 cases of oral teratoma, and 13 cases of epignathus.^8^ Case reports have been reported occasionally in Japan, shown in this report, there also others in Japan. Table 1 shows the summary of the cases where tracheotomy using the EXIT procedure has been implemented recently.^4,5,9–14^

The cases where fetal tracheotomy was performed often experienced the tracheal intubation, which gives enough time and makes the identification of the tracheal position relatively easy. However, the patient who receives the EXIT procedure are newborn babies. So, it is difficult to examine with the hand the ring cartilage and the wind pipes which are important parts for tracheotomy. Furthermore, no tracheal intubation makes the identification of the tracheal position difficult, while time constraint against the risk of low oxygen makes the difficulty level of the technique extremely high for operators.

Problems of the EXIT procedure includes, 1) the uterus must be relaxed to restrain the separation of the placental environment, 2) the longer the operation requires, the more bleeding occurs, 3) a greater burden is placed on the mother. Therefore, indications of the methos should be judged carefully. In addition, since the fetuses with the indication of the EXIT procedure often have various associated malformations, the procedure must be fully explained to family members of the affected fetuses and informed consent aquired consensus.^15^

When fetal cervical tumors and masses became giant, they are often said to be accompanied by a delivery disorder,^14^ and the most significant clinical problem is the polyhydramnios and respiratory disorder caused by the compression of the airway and the esophagus. Mirose et al. suggested that if the affected fetus has produced dysphagia caused by the esophagus compression due to tumors and is found to have polyhydramnios, the selection of EXIT procedure be considred.^2^ Now, in our case study, after the full explanation, including risks caused by airway compression due to fetal tumors and the operation, has been done, we decided to implement the EXIT procedure with the consent of all the family members.

The EXIT procedure cannot be successful without cooperation of multiple departments. The anesthetists are responsible for the stabilization of circulatory dynamics of the mother and the maintenance of uterine contraction until the extraction of the fetus. The obstetrician performs the caesarean operation as well as the fixation of fetal head and body trunk during conducting the EXIT procedure to maintain the body position. The obstetrician also simultaneously confirms the fetal condition by continually observing the fetal heartbeat, the blood circulation through the umbilical cord, and the placenta condition with the monitoring of the fetus during the surgery and using the fetal ultrasonic examination. The pedologist performs the tracheal intubation and the fetal management after the airway management. Thus, it becomes very important for the related departments and the relevant personnel to play their appropriate roles in close cooperation with one another. In our case study, conferences were held with the practitioners of the related department and also with multiple related professions. In respective conferences, the participants shared the information on the present method, set the target period, and examined the arrangement of the personnel, the operative position of the mother, the method for monitoring the fetus, the method for fixing the tube to the fetus after insertion of the tube, and the method of fixation of the fetal head when the tracheotomy was decided. We judged that tracheotomy was able to implement by peeling and tract the cystic masses covering the anterior cervix which is highly auspicious of cystic lymphangioma based on the image diagnosis. Further, it was conceivable that the tracheotomy under the present procedure was able to be completed appropriately as a result of thorough simulations using the operating room.

In general, the limitation to the EXIT procedure is thought to be around one hour,^4^ the average time for the EXIT procedure is 28.5 minutes, but the case with the longest time of 157 minutes for teratoma was reported.^8^ In our case study, the operating time, a factor which affects the mother and her fetus, was 31 minutes, and it was almost equivalent to the target time set in advance.

There were a large number of cases where the tracheal intubation achieved airway management,^8,10^ while skillful anesthetists and pedologists might also have difficulty in tracheal intubation. If the frequency of intubation increases, intubation will become impossible due to bleeding or laryngeal edemas, so that airway management using the tracheotomy will be required. This time, we set the upper limit of the intubation to 20 minutes in consideration of the maintenance time for blood flowing /oxygen supply through the umbilical cord, but we used the maximum time. So, the tracheal intubation was required to be done within 10 minutes in order to achieve the target time. In our case study, it was possible to conduct the tracheotomy in 11 minutes, but some operations may require longer significantly time depending on the conditions of the cervix and tumors.

Although we were not able to collect a wide range of literature which discusses time regulation, we considered that the following plan may be also helpful: when the frequency of intubation but not time is used to delimit timing between the intubation and tracheotomy, and if the intubation is impossible, the strategy should be shifted to the tracheotomy.

A small number of case reports have descried the implementation of the tracheotomy under the EXIT procedure, but this will serve as an extremely useful life-saving measure for the fetus associated with upper airway occlusion. With the increase of accuracy of recent imaging examination, various types of prenatal diagnosis have been made available, and the same response may be increasingly required in these cases. In the future, the EXIT procedure is expected to be widely recognized as a useful treatment for fetal airway management, widely recognized by embryonic airway management as a useful therapy, while the indication of tracheotomy will be evaluated using imaging diagnosis by an otolaryngologist, and the appropriate operation will be implemented as a continuation of infant tracheotomy.

## Conclusion

The tracheotomy using the EXIT procedure was implemented for the fetus suspected of having upper airway occlusion immediately after birth caused by a cervical cystic lymphangioma. Thorough conferences and simulations with other departments and multiple professions were conducted before the surgery with continuous detailed cooperation with one another. As a result, safe airway maintenance was made possible for the fetus with difficulty in tracheal intubation.

## Figures and Tables

**Table1:** 

Reported by:	Journals	Airway management	Prenatal diagnosis	Complication of congenital abnormalities	Life-saving just after birth	Final outcome
Fukuda	Nippon Jibiinkoka Gakkai Kaiho 2006	Tracheotomy	Teratoma	Unknown	Survival	Unknown

Tsujimura, et al.	J. Jpn. Bronchoesophagol 2011	Tracheotomy	1^st^ and 2^nd^ branchial arch syndromes	None	Survival	Survival

Morimoto	Nippon Jibiinkoka Gakkai Kaiho 2012	Tracheotomy	Teratoma	Unknown	Survival	Survival

Minami, et al.	J. Jpn. Bronchoesophagol. Soc. 2012	Tracheotomy	Cervical lymphoma	Unknown	Survival	Unknown

Adachi	Otologia Fukuoka 2014	Tracheotomy	Small forehead	Unknown	Survival	Unknown

Hongo, et al.	Iwate Igaku Zasshi 2018	Tracheotomy	Congenital high airway obstruction syndrome	Unknown	Survival	Unknown

Shobatake, et al.	J. Jpn. Soc. Pediatr. Surg 2019	Tracheotomy	Congenital high airway obstruction syndrome	None	Survival	Survival

			Congenital high airway obstruction syndrome	None	Survival	Survival

			Congenital high airway obstruction syndrome	Cornelia de Lange Syndrome Tracheomalaci Atrial septal defect	Survival	Death

			Congenital high airway obstruction syndrome	Bronchostenosis Duodenal atresia Aproctia	Survival	Death

Shobatake	Japanese Journal of Pediatric Hematology/Oncology 2019	Tracheotomy	Epignathus	None	Survival	Survival

			Epignathus	Unknown	Survival	Death

## Supplementary Material

PDF-Japanese
